# Genome-wide association and differential expression analysis of salt tolerance in *Gossypium hirsutum* L at the germination stage

**DOI:** 10.1186/s12870-019-1989-2

**Published:** 2019-09-11

**Authors:** Yanchao Yuan, Huixian Xing, Wenguan Zeng, Jialing Xu, Lili Mao, Liyuan Wang, Wei Feng, Jincai Tao, Haoran Wang, Haijun Zhang, Qingkang Wang, Guihua Zhang, Xianliang Song, Xue-Zhen Sun

**Affiliations:** 1 State Key Laboratory of Crop Biology/Agronomy College, Shandong Agricultural University, Taian, Shandong China; 2 College of Life Sciences, Qingdao Agricultural University, Key Lab of Plant Biotechnology in Universities of Shandong Province, Changcheng Road 700, Qingdao, China; 3 Heze Academy of Agricultural Sciences, Heze, China

**Keywords:** Salinity, Transcriptome, Genetic variation, Genome-wide association study, Germination stage, Comprehensive evaluation, Single-nucleotide polymorphisms (SNPs)

## Abstract

**Background:**

Salinity is a major abiotic stress seriously hindering crop yield. Development and utilization of tolerant varieties is the most economical way to address soil salinity. Upland cotton is a major fiber crop and pioneer plant on saline soil and thus its genetic architecture underlying salt tolerance should be extensively explored.

**Results:**

In this study, genome-wide association analysis and RNA sequencing were employed to detect salt-tolerant qualitative-trait loci (QTLs) and candidate genes in 196 upland cotton genotypes at the germination stage. Using comprehensive evaluation values of salt tolerance in four environments, we identified 33 significant single-nucleotide polymorphisms (SNPs), including 17 and 7 SNPs under at least two and four environments, respectively. The 17 stable SNPs were located within or near 98 candidate genes in 13 QTLs, including 35 genes that were functionally annotated to be involved in salt stress responses. RNA-seq analysis indicated that among the 98 candidate genes, 13 were stably differentially expressed. Furthermore, 12 of the 13 candidate genes were verified by qRT-PCR. RNA-seq analysis detected 6640, 3878, and 6462 differentially expressed genes at three sampling time points, of which 869 were shared.

**Conclusions:**

These results, including the elite cotton accessions with accurate salt tolerance evaluation, the significant SNP markers, the candidate genes, and the salt-tolerant pathways, could improve our understanding of the molecular regulatory mechanisms under salt stress tolerance and genetic manipulation for cotton improvement.

**Electronic supplementary material:**

The online version of this article (10.1186/s12870-019-1989-2) contains supplementary material, which is available to authorized users.

## Background

Salinity is a significant abiotic stress that reduces crop productivity and quality throughout the world [1]. More than 6% of the world’s 800 million agricultural lands are affected by salinity [2]. A comprehensive understanding of the salt-responsive molecular mechanisms and exploring salt-tolerant genes will help increase crop tolerance to salinity [1, 3, 4]. Upland cotton (*Gossypium* spp.) is an important source of natural fiber, vegetable oil, and protein and is also a moderately salt-tolerant and pioneer crop that can be grown in saline-alkali land. Nevertheless, its yield will be drastically reduced as the soil salinization level increases [5]. Tolerance to salinity significantly varies among cotton germplasms. Thus, screening elite high salt-tolerant germplasms is key to breeding salt-tolerant cotton, as well as in identifying molecular mechanisms and key genes associated with salt tolerance.

Several methods have been developed for assessing germplasm. Factor analysis of principal component analysis (PCA) is commonly used in the evaluation of the status of each material in a group by analyzing a large number of samples and major correlation indicators [6–9]. Subordinate function analysis (SFA) is often used to evaluate stress tolerance [8, 10]. However, the evaluation may be one-sided when using only PCA or SFA [10, 11]. A comprehensive evaluation value combining PCA and SFA can convert each indicator into independent factors that can be compared with each other while maintaining the original information, thereby providing a more comprehensive assessment of plant tolerance [10]. In addition, this comprehensive evaluation value has higher accuracy and efficiency than grade evaluation with estimating intuitive withered area proportion. This integrated approach has been used to assess stress tolerance in sugarcane [10, 12], cucumber [13], tomato [11], alfalfa [8], wheat [14], and cotton [15].

Salinity tolerance is a multigene controlled trait and is susceptible to environmental factors. Association mapping based on linkage disequilibrium (LD) is a common and powerful technique for identifying genomic regions related to specific variants of phenotypic characteristics [16] based on its capability of dissecting a larger number of alleles than linkage mapping. However, studies on identifying salt-tolerant QTLs/genes in cotton using association mapping [7, 16, 17] or even linkage mapping [18, 19] are limited. In addition, the results of a single investigation on association mapping for salt tolerance in cotton using high-density single-nucleotide polymorphism (SNP) markers have been reported. A total of 23 significant SNPs and 280 possible candidate genes, of which most are involved with transcription factors, transporters, and enzymes, were found to be associated with two salt tolerance-related traits [20]. With the maturity and popularization of second-generation sequencing, RNA sequencing (RNA-seq) has become the major approach in excavating candidate genes, as well as in constructing molecular regulatory pathways and potential regulatory networks. Some salt-responsive mRNAs [21, 22], miRNAs [3], alternative splicing [23], or long non-coding RNAs (lncRNAs) [24] were detected in cotton using RNA-seq and the potential molecular regulatory pathways or regulatory networks of some genes were preliminarily explored. In cotton, several salt stress-inducible genes have been detected through association or linkage mapping and RNA-seq, including *GhNHX1* [25], metallothionein (*GhMT3a*) [26], *GhERF2-GhERF6* [27, 28], *GhDREB1* [29], CCCH-type zinc finger (*GhZFP1*) [30], *GhNAC1-GhNAC631* [31], *GhMPK2* [32], *GhMKK1* [33], *GhSOD1* and *GhCAT1* [34], *GhWRKY17* [35], and *GhAnn1* [36].

In this study, we combined comprehensive evaluation, association mapping, and RNA-seq to explore salt-tolerant candidate chromosomal regions/genes in cotton at the germination stage. This study provides candidate QTL (qualitative-trait locus) regions and genes for dissecting the genetic mechanisms of salt tolerance and variety breeding in cotton.

## Results

### Salt tolerance performance and evaluation

At germination stage, 10 traits were measured in 196 upland cotton genotypes, and the results are shown in Table 1. The results indicated that all 10 traits were significantly hindered by salt stress. All traits exhibited significantly lower means and extreme values under salt stress than the normal conditions. Comparatively, GP (germination potential) was the most affected trait, whereas SFM (shoot fresh mass) was least affected. The 0.3% (200 mmol/L) NaCl stress reduced not only the total germination rate but also the germination speed and subsequent growth. Under normal conditions, the CVs (coefficient variations) ranged from 7.1% in RDM (root dry mass) to 20.6% in VI (vigor index). The 0.3% (200 mmol/L) NaCl stress resulted in significantly increased CVs in nearly all 10 traits, except for VI and RFM (root fresh mass). SDM (shoot dry mass), GP, and GR (germination rate) ranked the top three CVs. These results indicated that most of the trait responses of this panel of upland cotton to salt stress are highly diverse.

**Table 1:** Statistics and difference analysis of traits related to salt tolerance under salt stress and non-salt stress

Trait	GR (%)		GP (%)		GI		VI		RL (cm)		RDM (mg)		RFM (mg)		PH (cm)		SDM (mg)		SFM (mg)	
	CK	Salt	CK	Salt	CK	Salt	CK	Salt	CK	Salt	CK	Salt	CK	Salt	CK	Salt	CK	Salt	CK	Salt
Min	90.0	23.0	75.0	0.0	58.5	9.3	92.6	3.9	7.8	3.0	201.0	10.0	412.0	40.0	7.9	2.9	231.0	20.0	1080.0	220.0
Max	100.0	87.0	100.0	74.0	63.5	53.0	103.3	60.5	9.7	7.1	240.0	120.1	450.0	230.0	12.6	8.6	260.0	160.0	1630.0	890.0
Mean	91.0	45.3	82.1	21.5	53.1	37.0	45.9	32.0	8.5	3.1	221.2	70.0	210.0	73.5	10.7	5.1	240.0	98.0	870.0	603.0
SD	7.7	13.0	9.9	6.8	6.7	8.8	9.5	5.7	0.9	0.4	15.8	9.3	0.0	10.3	0.9	0.8	0.0	35.3	0.1	90.6
CV(%)	8.5	28.8	12.0	27.9	12.7	23.7	20.6	17.8	11.0	12.2	7.1	13.3	15.2	14.0	8.7	15.1	13.6	36.0	13.2	15.0
t-test	56.248**		30.778**		37.452**		43.032**		22.774**		1.439**		4.716**		51.944**		33.782**		38.912**	

**indicate significant difference at *p* < 0.01

To reduce the impact of environmental factors and to reach high evaluation accuracy, the BLUPed (BLUP: the best linear unbiased prediction) STI (salt tolerance index) was calculated based on the nine (three years × three replications) STIs in each trait, and the results were shown in Additional file 1: Table S1 [37, 38]. No significant differences among STIs among the 3 years and the BLUPed STIs of each trait were observed. However, the BLUPed STIs and their CVs significantly varied among traits. The GR, RL (root length), and PH (plant height) exhibited larger BLUPed STIs with smaller CVs, whereas VI showed the smallest BLUPed STIs with large CVs. GP and SFM had small BLUPed STIs with larger CVs. The traits that had high salt tolerance (high BLUPed STI) usually had low CVs and vice versa, except for SDM, which exhibited the second smallest BLUPed STI with medium CV value. To further reveal the relationship among the measured traits under salt stress, Pearson correlation coefficients were estimated and are listed in Additional file 2: Table S2. Significant positive correlations were detected among nearly all trait-pairs except for RFM-SDM and RFM-SFM, which exhibited very weak and insignificant correlations.

### Comprehensive evaluation of salt tolerance

To comprehensively evaluate salt tolerance in this panel, PCA of STIs was performed. Before PCA, the Kaiser-Mayer-Olkin (KMO) measure and Bartlett’s test were adopted to check whether the STIs were suitable for factor analysis. The KMO values in the four data sets (2014: 0.825; 2015: 0.856; 2016: 0.852; and BLUP: 0.857) were all > 0.5, and the Bartlett’s test values all performed well (χ^2^ > χ^2^_0.01, 45_, and Sig. = 0.000 < 0.05) [6], indicating that the STI data were suitable for PCA. The four STI data sets resulted in consistent results in PCA. For each set of STI data, two principal components that accounted for at least 86.85% of the total variance were obtained based on the Eigenvalues-more-than-1.0 rule (Additional file 3: Figure S1 and Additional file 4: Table S3). As shown in PCA plots (Additional file 5: Figure S2), Factor 1 represents all the salt-tolerance traits except for STI_RFM (salt tolerance index of root fresh mass), which was Factor 2.

Furthermore, the comprehensive evaluation value (D) of each genotype was obtained based on the subordinate function value (U) computed with the two principle factors (Additional file 6: Table S4). By K-mean cluster analysis, the 196 genotypes were divided into four groups based on the four sets of D values, which represented the salt tolerance of genotypes in 2014, 2015, 2016, and BLUPed data. Of the 196 genotypes, 27 accessions ranked as advanced salt-tolerant with D values for BLUPed STI within the range of 0.669–0.934, 35 accessions had medium salt tolerance, with D values ranging from 0.486 to 0.633), 67 were salt-sensitive with D values from 0.320 to 0.474, and 67 ranked as high salt-sensitive with D values from 0.053 to 0.312 (Additional file 6: Table S4).

### Association of SNP markers and salt tolerance

**Fig. 1:**
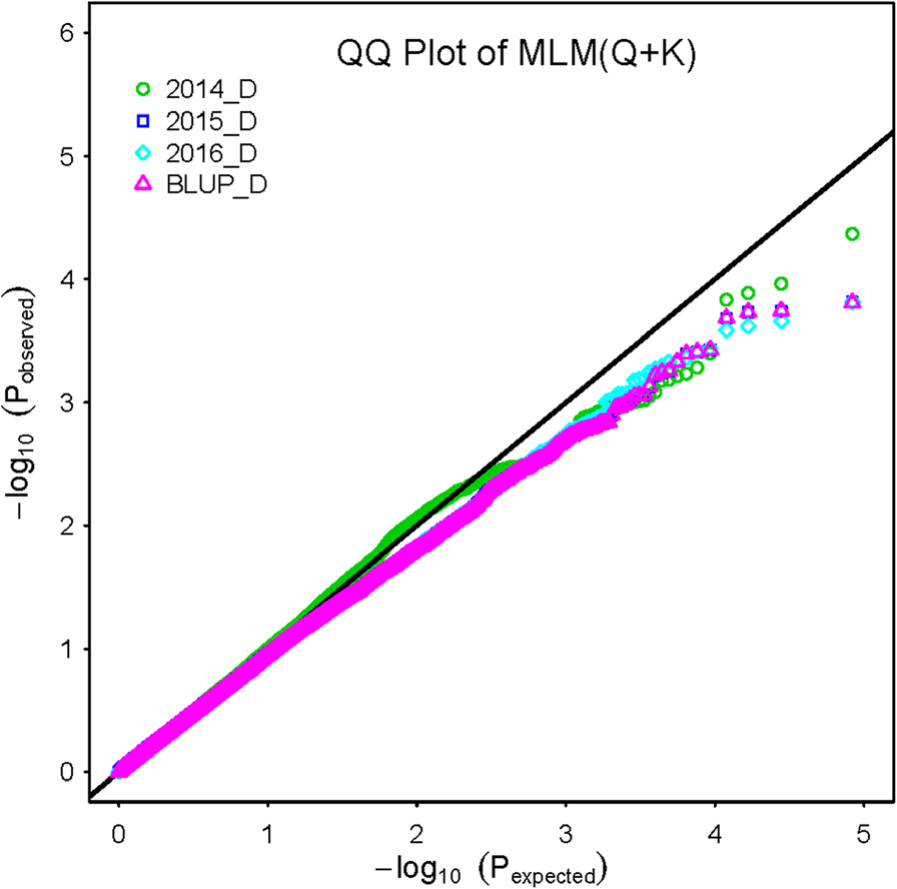
Q-Q plots for D values in 2014, 2015, 2016, and BLUPs using MLM (Q + K)

**Fig. 2:**
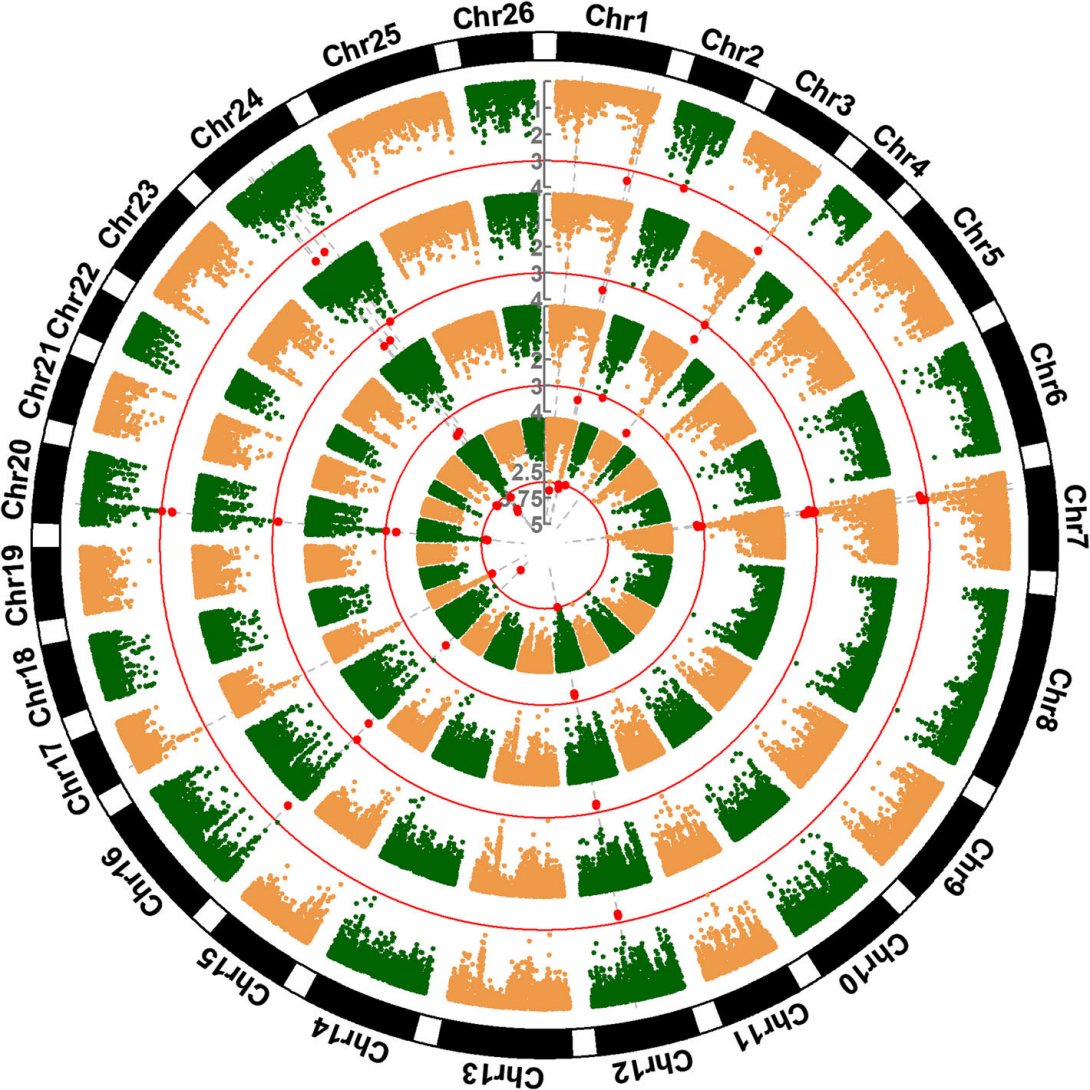
Circular-Manhattan plot for D values GWAS results. The threshold value was set at *p* < 0.001

In this GWAS (genome-wide association study), the comprehensive evaluation values (D) was used as phenotypic data to detect significant salt tolerance QTLs/genes. Thus, the covariant Q matrix, which can reduce the negative influence of group structure, was introduced into the mixed linear model (MLM) to reduce false positives. To test the quality of the mixed linear model combined structure Q matrix and kinship matrix (MLM [Q + K]) and association results, we employed a QQ (Quantile quantile) plot. Figure 1 showed that the MLM [Q + K] model was slightly too strict in detecting significant SNP markers, indicating that the probability of false positives was much lower. Consequently, a total of 33 SNPs that were significantly (*p* < 0.001) associated with salt tolerance were detected using MLM [Q + K] (Fig. 2 and Table 2). Among these, 17 SNPs were detected in at least two environments (3 years and BLUPed data) and 7 SNPs were identified in all four environments, explaining 5.98 to 10.76% phenotypic variation, with an average of 8.21%. These 17 SNPs were consequently used to identify candidate QTLs. When the distance between the first SNP and neighboring SNPs was less than the LD decay distance at pairwise r^2^ = 0.1 level on each chromosome (Additional file 7: Table S5) or r^2^ between the first SNP and the neighboring SNP was > 0.1, these SNP markers were regarded as a confidence interval for a candidate QTL. Finally, 13 QTLs were obtained on 10 chromosomes, including 4 QTLs on homologous chromosomes A07–D07.

**Table 2:** Significant SNP markers, QTLs, and candidate genes related to salt tolerance

Marker	Chr	Position (bp)	-lg(P) (BLUP)	MarkerR^2^ (BLUP)	Environments	QTL	Physical Chr	Genomic position	No. of genes
TM3300	1	92,501,634	3.41	0.084	BLUP, 14, 15, 16	*qGhST-c1*	A01	92,402,641..92562754	7
TM4974	2	56,676,599	3.05	0.079	BLUP, 15	*qGhST-c2*	A02	56,176,476..56990091	6
TM8361	3	98,536,836	3.81	0.108	BLUP, 15, 16	*qGhST-c3*	A03	98,507,289..98618065	13
TM18816	7	8,674,003	3.26	0.083	BLUP, 15, 16	*qGhST-c7–1*	A07	8,654,507..8730789	5
TM19026	7	11,292,696	3.22	0.090	BLUP, 15, 16	*qGhST-c7–2*	A07	11,270,123..11494991	12
TM19028	7	11,312,723	3.06	0.087	BLUP, 15, 16		A07		
TM19030	7	11,334,462	3.06	0.080	BLUP, 15, 16		A07		
TM19035	7	11,356,480	3.13	0.084	BLUP, 15, 16		A07		
TM41811	12	57,971,233	3.25	0.065	BLUP, 15, 16	*qGhST-c12–1*	A12	57,740,976..58054684	11
TM41814	12	58,100,057	3.33	0.069	BLUP, 14, 15, 16	*qGhST-c12–2*	A12	58,097,661..58230406	3
TM63245	16	2,688,021	3.00	0.060	BLUP, 16	*qGhST-c16–1*	D07	2,555,918..2766294	27
TM63387	16	5,545,380	3.73	0.099	BLUP, 14, 15, 16	*qGhST-c16–2*	D07	5,518,153..5573449	3
TM73567	20	6,921,040	3.43	0.068	BLUP, 14, 15, 16	*qGhST-c20–1*	D10	6,887,955..6965236	7
TM73573	20	6,959,800	3.02	0.077	BLUP, 15		D10		
TM73579	20	6,996,881	3.40	0.068	BLUP, 14,15, 16	*qGhST-c20–2*	D10	6,991,910..7032029	2
TM67763	24	16,771,670	3.74	0.099	BLUP, 14, 15, 16	*qGhST-c24–1*	D08	16,766,258..16776082	0
TM68258	24	19,887,759	3.68	0.096	BLUP, 14, 15, 16	*qGhST-c24–2*	D08	19,884,918..19901251	2
TM3510	1	96,629,867	3.24	0.092	14				
TM776	1	14,254,094	3.40	0.098	14				
TM4108	2	7,344,966	3.00	0.082	14				
TM8353	3	98,481,949	3.01	0.079	16				
TM18851	7	9,689,745	3.05	0.084	16				
TM18989	7	11,034,281	3.07	0.081	16				
TM19015	7	11,198,513	3.01	0.078	16				
TM19017	7	11,226,724	3.09	0.080	16				
TM19018	7	11,232,229	3.07	0.079	16				
TM19023	7	11,276,537	3.20	0.082	16				
TM53602	17	4,200,687	3.18	0.092	14				
TM71972	23	33,356,265	3.02	0.064	14				
TM71987	23	33,461,358	3.09	0.066	14				
TM67849	24	17,321,002	3.89	0.112	14				
TM68645	24	31,157,957	3.21	0.089	14				
TM68879	24	44,114,822	3.03	0.060	16				

### Candidate genes detected with GWAS

According to the physical positions of the candidate QTLs referenced to the *G. hirsutum* (TM-1) genome [39], a total of 98 candidate genes were detected (Table 2), and all these genes were annotated in *Arabidopsis thaliana* (Additional file 8: Table S6). The candidate genes were retrieved in UniProtKB to annotate their biological processes (Additional file 8: Table S6). Functional annotation showed that 35 candidate genes were associated with salt tolerance (Table 3). Of these, 11 genes were involved in eight transcription factor (TF) families, including CO-like, MYB, bZIP, ERF, TALE, SBP, HD-ZIP, and ARR-B; 17 genes were related to “response to stress” or “defense response” such as the stress of acidic pH, heat, hydrogen peroxide, water deprivation, and hyperosmotic salinity; nine genes were associated with “signaling” or “response to signal factors” such as salicylic acid (SA), gibberellic acid (GA), jasmonic acid (JA), calcium-mediated signaling, cytokinin, and abscisic acid (ABA); six genes were involved in “ion homeostasis” or “ion transport”, one gene was associated with “fatty acid biosynthetic process”, and five genes were related to amino acid synthesis or transport. In addition, 12 of these 35 candidate genes were involved in two or three functional categories (Table 3).

**Table 3:** Candidate genes belong to categories related to salt tolerance

Category	Gene ID	Gene Name	Description	TF Family/Stress/Signal/Ion transport
Transcription factors	Gh_A01G1562	COL16	Zinc finger protein CONSTANS-LIKE 16	CO-like
	Gh_A01G1564	MYB108	Transcription factor MYB108	MYB
	Gh_A03G1731	BZIP53	bZIP transcription factor 53	bZIP
	Gh_A03G1738	BLH6	BEL1-like homeodomain protein 6	TALE
	Gh_A12G0867	SPL8	Squamosa promoter-binding-like protein 8	SBP
	Gh_A12G0870	ERF034	Ethylene-responsive transcription factor ERF034	ERF
	Gh_A12G0871	HAT3	Homeobox-leucine zipper protein HAT3	HD-ZIP
	Gh_A12G0875	ERF13	Ethylene-responsive transcription factor 13	ERF
	Gh_A12G0876	ERF13	Ethylene-responsive transcription factor 13	ERF
	Gh_A12G0877	ERF2	Ethylene-responsive transcription factor 2	ERF
	Gh_D07G0251	ARR12	Two-component response regulator ARR12	ARR-B
Response to stress or defense response	Gh_A01G1563	CUT1	3-ketoacyl-CoA synthase 6	Cold
	Gh_A03G1732	AT2G16385	CIF1:Proteincasparlan strip integrity factor 1	Acidic pH
	Gh_A03G1733	AT4G39130	Late embryogenesis abundant protein	Stress, Water
	Gh_A07G0622	CLPB1	Chaperone protein ClpB1	Heat, Hydrogen peroxide
	Gh_A07G0624	DGK1	Diacylglycerol kinase 1	Defense response
	Gh_A12G0875	ERF13	Ethylene-responsive transcription factor 13	Defense response
	Gh_A12G0876	ERF13	Ethylene-responsive transcription factor 13	Defense response
	Gh_A12G0877	ERF2	Ethylene-responsive transcription factor 2	Defense response
	Gh_D07G0240	KAT3	Potassium channel KAT3	Nematode
	Gh_D07G0244	EDR2L	Protein enhanced disease resistance 2-like	Defense response
	Gh_D07G0246	CHIA	Acidic mammalian chitinase	Immune system process
	Gh_D07G0248	CHIT1	Chitotriosidase-1	Immune response
	Gh_D07G0249	CHIT1	Chitotriosidase-1	Immune response
	Gh_D07G0251	ARR12	Two-component response regulator ARR12	Water deprivation
	Gh_D07G0252	NKS1	Ubiquitin-associated protein (DUF1068)	Hyperosmotic salinity
	Gh_D07G0256	TIL	Temperature-induced lipocalin-1	Hyperosmotic salinity, Cold, Freezing, Heat, Paraquat, Water deprivation
	Gh_D07G0500	HVA22E	HVA22-like protein e	Hyperosmotic salinity, Cold, Water deprivation
Signaling or response to signal factors	Gh_A01G1564	MYB108	Transcription factor MYB108	Gibberellic acid, Jasmonic acid
	Gh_A02G1099	WAK2	Wall-associated receptor kinase 2	Salicylic acid
	Gh_A02G1100	WAK2	Wall-associated receptor kinase 2	Salicylic acid
	Gh_A07G0624	DGK1	Diacylglycerol kinase 1	Intracellular signal transduction
	Gh_A07G0729	RALFL27	Protein RALF-like 27	Calcium mediated signaling, Cell-cell signaling
	Gh_D07G0251	ARR12	Two-component response regulator ARR12	Cytokinin
	Gh_D07G0256	TIL	Temperature-induced lipocalin-1	Cytokinin
	Gh_D07G0500	HVA22E	HVA22-like protein e	Abscisic acid
	Gh_D10G0643	IAA27	Auxin-responsive protein IAA27	Auxin
Ion homeostasis or ion transport	Gh_A03G1732	AT2G16385	CIF1:Proteincasparlan strip integrity factor 1	Ion homeostasis
	Gh_D07G0240	KAT3	Potassium channel KAT3	Potassium ion transmembrane transport, Regulation of ion transmembrane transport, Regulation of membrane potential
	Gh_D07G0252	NKS1	Ubiquitin-associated protein (DUF1068)	Vacuolar sequestering of sodium ion
	Gh_D07G0254	MOT1	Molybdate transporter 1	Molybdate ion transmembrane transporter activity, Molybdate ion transport
	Gh_D07G0255	MOT1	Molybdate transporter 1	Molybdate ion transmembrane transporter activity, Molybdate ion transport
	Gh_D07G0256	TIL	Temperature-induced lipocalin-1	Cellular chloride ion homeostasis, Cellular sodium ion homeostasis
Fatty acid	Gh_A01G1563	CUT1	3-ketoacyl-CoA synthase 6	Fatty acid biosynthetic process
Amino acid	Gh_D07G0242	PROC	Pyrroline-5-carboxylate reductase	Proline biosynthetic process, Pyrroline-5-carboxylate reductase activity
	Gh_A03G1734	HISN7	Bifunctional phosphatase IMPL2, chloroplastic	Histidine biosynthetic process, Inositol biosynthetic process, Phosphatidylinositol phosphorylation
	Gh_A03G1737	SAE2	SUMO-activating enzyme subunit 2	Acid-amino acid ligase activity
	Gh_D08G0928	GDU2	Protein GLUTAMINE DUMPER 2	Amino acid transport
	Gh_D08G0929	GDU3	Protein GLUTAMINE DUMPER 3	Amino acid transport

**Fig. 3:**
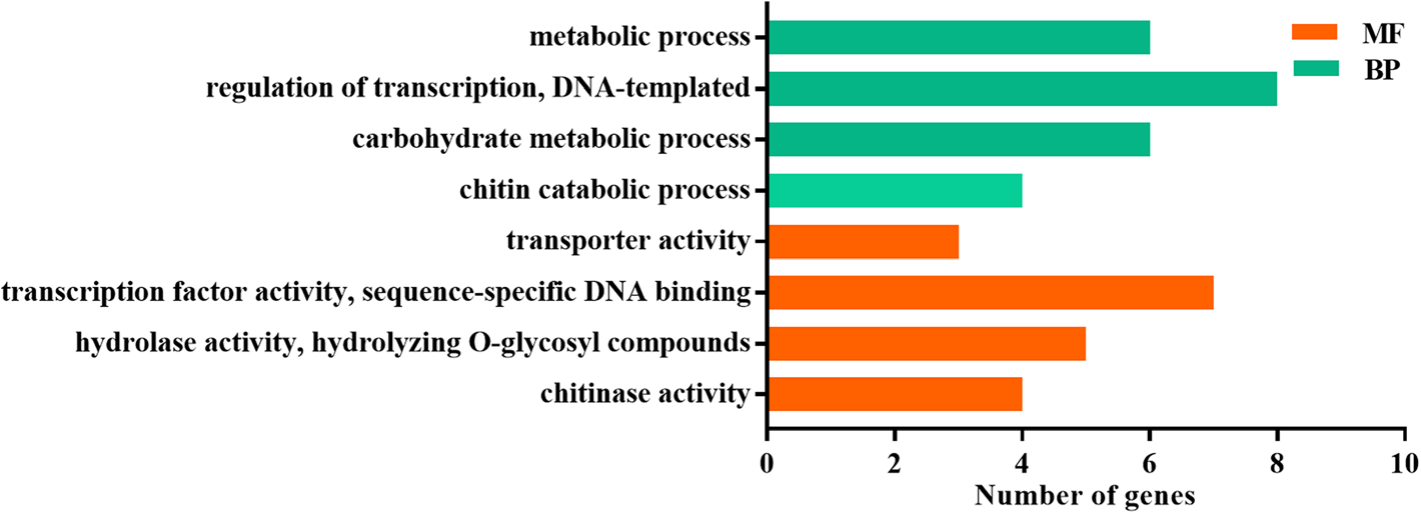
Gene Ontology (GO) enrichment analysis of 98 salt-response genes detected in GWAS

GO (Gene Ontology) enrichment analysis was conducted to further infer the functions of these 98 candidate genes. At a *P*-value < 0.05 and the number of genes > 3, the 98 candidate genes were categorized into eight GO terms (Fig. 3). Each of the two main categories, namely, biological process (BP) and molecular function (MF), contained four GO terms. The BP category in this study contained “chitin catabolic process”, “carbohydrate metabolic process”, and “metabolic process and regulation of transcription”, whereas the MF category contained “chitinase activity”, “hydrolase activity”, and “transcription factor activity and transporter activity”.

Based on the physical positions, 21 of the 98 candidate genes detected in the GWAS were proximal to significant SNPs, and six (Gh_A07G0622-TM18816, Gh_A07G0729-TM19028, Gh_A12G0874-TM41811, Gh_D07G0251-TM63245, Gh_D10G0640-TM73567, and Gh_D10G0647-TM73579) of them had significant SNPs in their coding sequences (CDS) (Additional file 9: Table S7). Furthermore, based on their functional annotation or biological process (Table 3), three (Gh_A07G0622, Gh_A07G0729, and Gh_D07G0251) of the six genes were related to the functional categories of “response to stress or defense response”, “signaling or response to signal factors” or “transcription factors”, implying their involvements in salt tolerance responses.

### Transcriptome sequencing

**Fig. 4:**
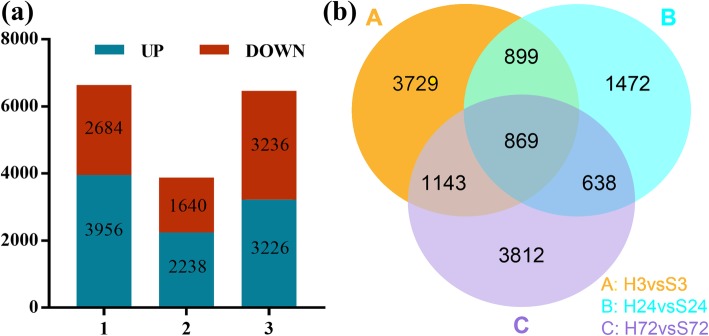
Statistical analysis of DEGs in the roots between salt stress and water control. **a** The number of upregulated and downregulated DEGs at different time points. **b** Venn diagram of DEGs at different time points after treatment

Root tissues of Han682 at the germination stage under 0.3% NaCl stress and normal conditions were harvested at 3, 24, and 72 h after treatment for RNA isolation. From the 12 RNA sequencing libraries of H3_1, H3_2, S3_1, S3_2, H24_1, H24_2, S24_1, S24_2, H72_1, H72_2, S72_1 and S72_2, a total of 50.5, 45.9, 46.6, 52.6, 46.8, 49.4, 45.1, 44.5, 43.6, 55.8, 45.3, and 40.6 million raw reads were obtained (Additional file 11: Table S8). At least 88% of the clean reads, which were raw reads without low-quality reads and adaptor sequences, were mapped to the *G. hirsutum* (TM-1) genome, in which uniquely mapped reads accounted for about 81% of the clean reads. A total of 6640, 3878, and 6462 DEGs (differentially expressed genes) (*P*-value < 0.05) were obtained between salt stress and the control at 3, 24, and 72 h post salt stress treatment, respectively, and 3956 (59.6%), 2238 (57.7%), and 3226 (49.9%) were upregulated (Fig. 4). Among the three sampling time points, we identified 869 shared DEGs, including 562 continuously upregulated and 307 continuously downregulated genes.

To verify our DEG results, qRT-PCR was adopted on 20 randomly selected DEGs, including 13 continuously upregulated and 7 downregulated genes (Additional file 10: Figure S3). Relative expression levels were calculated using the ΔΔCt method. The results of transcriptome sequencing coincided with our qRT-PCR findings.

**Fig. 5:**
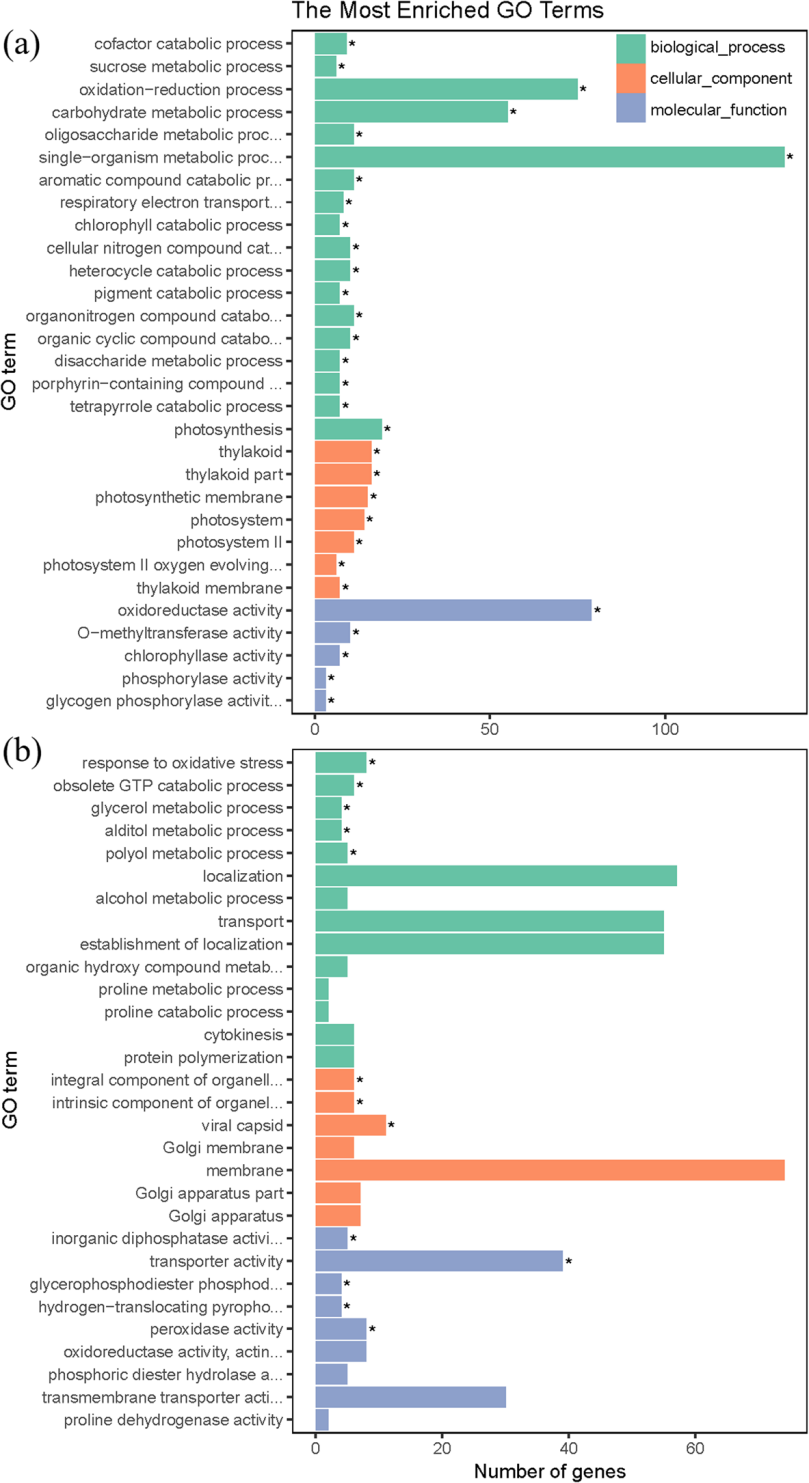
Gene Ontology (GO) functional enrichment analysis of salt-tolerance genes detected in RNA-seq. **a** GO functional classification of 562 continuously upregulated salt-tolerance genes at q-value < 0.05. **b** GO functional classification of 307 continuously downregulated salt tolerance genes at q-value < 0.05

To monitor salt tolerance gene expression, GO enrichment analysis (*P*-value corrected by FDR < 0.05) of 562 continuously upregulated genes and 307 continuously downregulated genes was conducted (Fig. 5). For continuously upregulated genes (Fig. 5a), the BP category consisted of “single-organism metabolic process”, “oxidation-reduction process”, “carbohydrate metabolic process”, “photosynthesis”, “organonitrogen compound catabolic process”, “oligosaccharide metabolic process” and “aromatic compound catabolic process”; the cellular component (CC) category was mainly related to “thylakoid” and “photosystem”; and the MF category comprised “oxidoreductase activity” and “O-methyltransferase activity”. For continuously downregulated genes (Fig. 5b), the BP category was mainly involved in “response to oxidative stress”, “obsolete GTP catabolic process”, and “polyol metabolic process”; the CC category was mainly involved in “viral capsid”, “integral component of organelle membrane”, and “intrinsic component of organelle membrane”; and the MF category was mainly involved in “transporter activity”, “peroxidase activity”, and “inorganic diphosphatase activity”. All the enriched GO terms of the 869 shared DEGs were associated with stress responses.

**Fig. 6:**
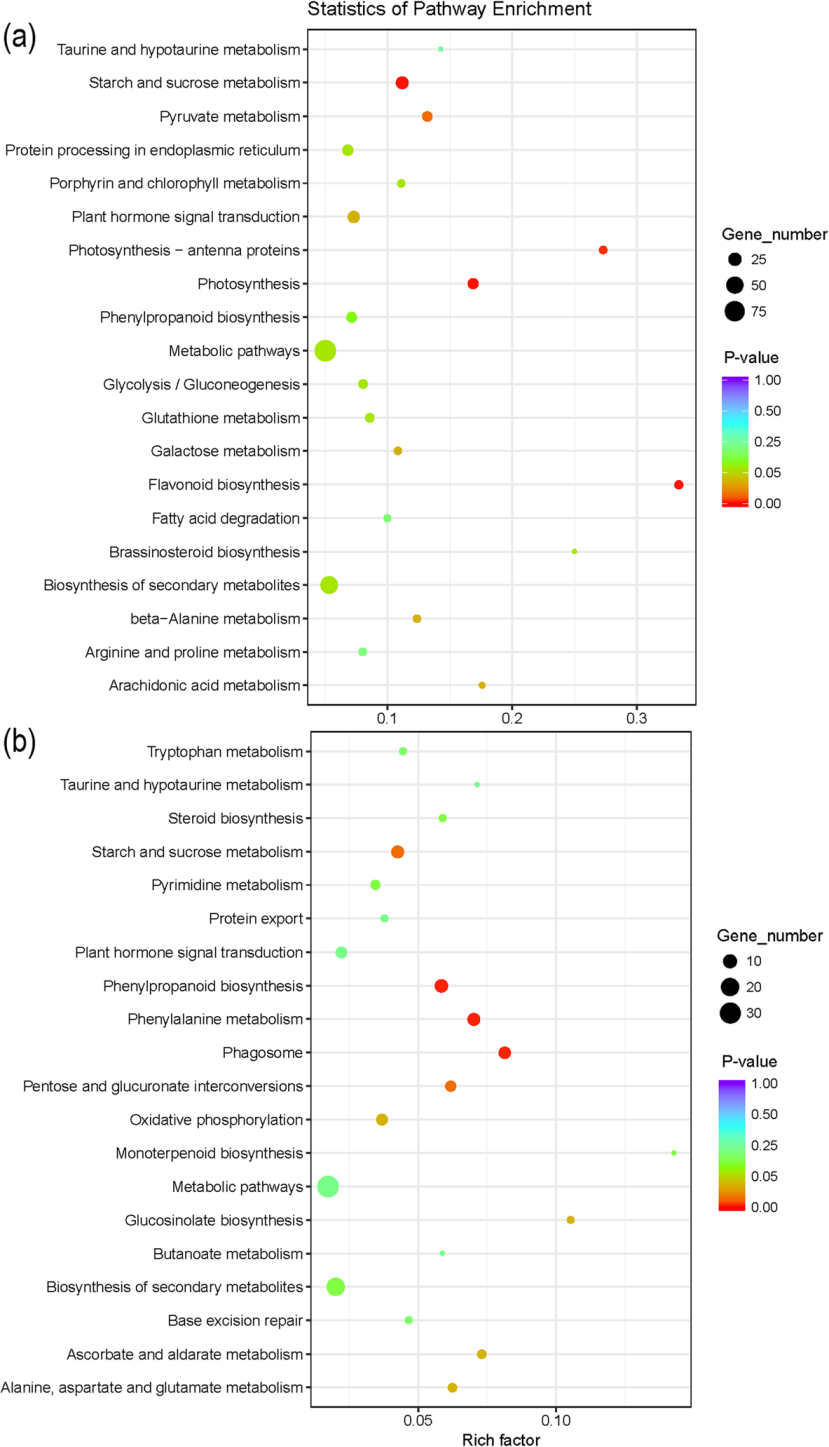
Statistics of pathway enrichment analysis of salt-tolerance genes detected in RNA-seq. **a** Pathway enrichment analysis of 562 continuously upregulated salt-tolerance genes at *p*-value < 0.05. **b** Pathway enrichment analysis of 307 continuously downregulated salt tolerance genes at *p*-value < 0.05

KEGG (Kyoto Encyclopedia of Genes and Genomes) pathway enrichment of the 869 continuously up/downregulated DEGs was performed (Fig. 6). The continuously upregulated genes were significantly (*P*-value < 0.05) enriched in nine KEGG pathways (Fig. 6a), including “photosynthesis”, “flavonoid biosynthesis”, “starch and sucrose metabolism”, “photosynthesis-antenna proteins”, “pyruvate metabolism”, “plant hormone signal transduction”, “beta-alanine metabolism”, “galactose metabolism”, and “arachidonic acid metabolism”. The KEGG pathways of continuously upregulated genes correspond to the GO enrichment results and the functions of genes, which were associated with stress response. The continuously downregulated genes were mostly related to nine KEGG pathways (Fig. 6b), including phagosome, phenylalanine metabolism, phenylpropanoid biosynthesis, starch and sucrose metabolism, pentose and glucuronate interconversions, ascorbate and aldarate metabolism, glucosinolate biosynthesis, oxidative phosphorylation and alanine, and aspartate and glutamate metabolism.

TFs play pivotal roles in plant stress responses [40]. In this study, 62 (7.13%) genes encoding for TFs were identified as shared DEGs, of which 41 were continuously upregulated and 21 continuously downregulated (Table 4). All the 62 genes were belonged to 19 TF families, including several key regulatory gene families responding to abiotic and biotic stresses such as ARF, bHLH, bZIP, C2H2, ERF, HD-ZIP, MYB, MYB_related, NAC, and WRKY.

**Table 4:** Shared DEGs belonging to TF families

TF family	Total	Up	Down
bZIP	11	3	8
HD-ZIP	6	5	1
MYB	6	5	1
NAC	5	4	1
bHLH	4	2	2
MYB_related	3	2	1
WRKY	3	2	1
G2-like	2	1	1
HSF	4	4	0
C3H	3	3	0
CO-like	3	3	0
LBD	2	2	0
M-type_MADS	2	2	0
NF-YB	2	2	0
NF-YA	1	1	0
ARF	2	0	2
C2H2	1	0	1
ERF	1	0	1
TCP	1	0	1
Total	62	41	21

### Combination of association analysis and transcriptome sequencing

We combined the GWAS and RNA-Seq results to further screen salt tolerance candidate genes. Of the 98 candidate genes in GWAS, 13 exhibited significantly different expression levels (*p* < 0.005) at more than one sampling time point in RNA-seq analysis (Table 5). Of the 13 putative DEGs, eight exhibited significantly different expression at only one time point, four (Gh_A03G1740, Gh_A12G0877, Gh_D07G0263, and Gh_D07G0500) at two time points, and one, Gh_A07G0622, upregulated at all three time points. In addition, six of the 13 putative DEGs (Gh_A01G1563, Gh_A02G1100, Gh_A07G0622, Gh_A12G0877, Gh_D07G0251, and Gh_D07G0500) were proximal to significant SNPs in GWAS, and two (Gh_A07G0622 and Gh_D07G0251) of the six genes had significant SNPs within coding sequence regions (Additional file 9: Table S7). In addition, as shown in above, Gh_A07G0622 and Gh_D07G0251 were involved in “response to stress or defense response”, “signaling or response to signal factors”, and “transcription factors” (Table 3).

**Table 5:** The differential expression of the putative genes detected in both GWAS and transcriptome sequencing

Gene_id	readcount_H3	readcount_S3	log2FoldChange	*p* value	readcount_H24	readcount_S24	log2FoldChange	*p* value	readcount_H72	readcount_S72	log2FoldChange	*p* value
Gh_A01G1563	40.232	78.450	0.963	0.261	4.001	51.024	3.673	0.000*	0.000	5.109	+∞	0.316
Gh_A02G1100	4.161	26.612	2.677	0.001*	2.994	2.954	−0.020	1.000	5.117	1.099	−2.219	0.297
Gh_A03G1740	587.138	96.274	−2.608	0.000*	322.348	67.547	−2.255	0.000*	38.368	24.008	−0.676	0.246
Gh_A07G0622	180.915	912.736	2.335	0.000*	185.769	1681.857	3.178	0.000*	130.525	1972.522	3.918	0.000*
Gh_A07G0623	24.501	51.165	1.062	0.036	48.834	79.571	0.704	0.403	32.992	79.503	1.269	0.004*
Gh_A12G0877	10.051	4.262	−1.238	0.306	27.855	0.484	−5.848	0.000*	25.105	3.719	−2.755	0.002*
Gh_D07G0243	63.337	213.505	1.753	0.000*	115.184	114.475	− 0.009	0.990	88.638	69.300	−0.355	0.441
Gh_D07G0249	1.579	0.444	−1.830	0.771	4.501	3.006	−0.582	0.834	2.439	77.211	4.984	0.000*
Gh_D07G0250	3.196	39.993	3.645	0.004*	0.501	4.404	3.137	0.205	7.333	4.395	−0.738	0.621
Gh_D07G0251	193.354	458.100	1.244	0.019	189.755	242.694	0.355	0.564	192.524	376.097	0.966	0.002*
Gh_D07G0258	220.517	323.412	0.552	0.060	141.005	164.415	0.222	0.586	95.896	206.969	1.110	0.002*
Gh_D07G0263	41.671	152.914	1.876	0.000*	25.937	198.357	2.935	0.000*	279.158	178.035	−0.649	0.043
Gh_D07G0500	37.958	426.805	3.491	0.000*	37.418	163.250	2.125	0.000*	262.034	249.357	−0.072	0.846

*indicate significant at the *p* < 0.005 level

**Fig. 7:**
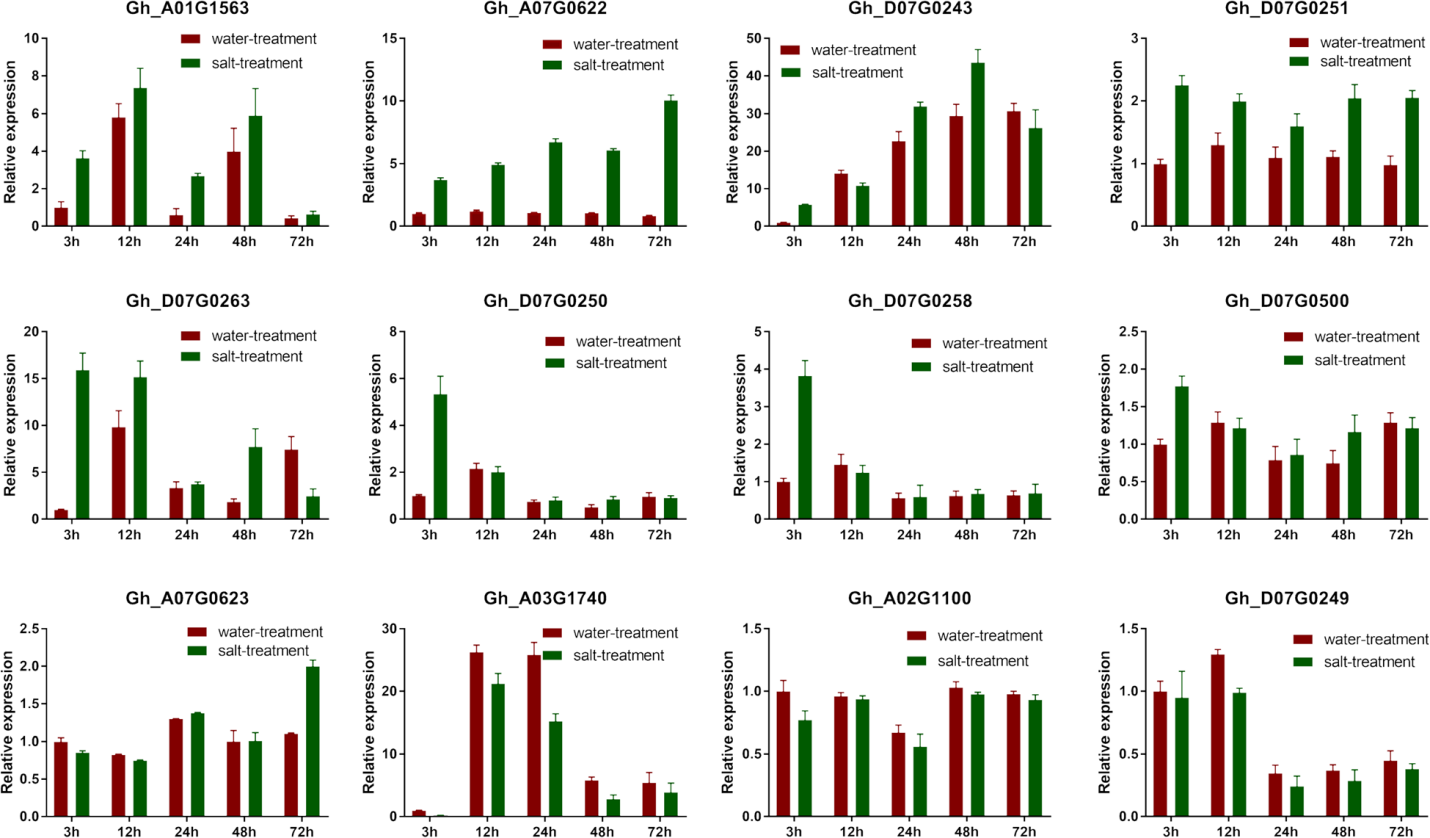
Quantitative real-time PCR validation of putative salt-responsive genes detected in combined analysis of GWAS and RNA-seq

To further verify the putative genes, all the 13 putative DEGs except for Gh_A12G0877, which could not be distinguished from a homologous gene, were analyzed by qRT-PCR using a set of three replicates for each sample at 3, 12, 24, 48, and 72 h after salt or water treatment. Relative expression levels were calculated using the ΔΔCt method. Figure 7 showed that the putative genes Gh_A01G1563, Gh_A07G0622, Gh_D07G0243, and Gh_D07G0251 were continuously upregulated after salt treatment. The genes Gh_D07G0623, Gh_D07G0250, Gh_D07G0258, and Gh_D07G0500 responded to salt stress at the initial stage (3 h or 12 h post treatment). The gene Gh_A07G0623 was responsive to salt at 72 h post treatment. The candidate genes Gh_A03G1740, Gh_A02G1100, and Gh_D07G0249 were continuously downregulated under salt stress. The qRT-PCR results showed all the 12 DEGs might be involved in salt tolerance responses.

## Discussion

### Comprehensive evaluation of salt tolerance

The salt tolerance traits of upland cotton are complex and vary with species, developmental stage, and tissue [41]. Thus, it is very important to evaluate germplasm salt tolerance accurately with a precise method. Several indexes have been used to assess salt tolerance in cotton, including indicators of seed germination (GR, GP, GI (germination index), and VI), plant morphological indexes (PH, SFM, SDM, and RL), physiological and biochemical indexes (Na^+^, K^+^, and betaine), and yield traits (boll number, boll weight, and lint yield) [7, 20, 42]. At present, it is generally believed that the comprehensive evaluation of salt tolerance combined with multi-indexes and multi-methods is more authentic and reliable such as PCA, SFA, and the comprehensive evaluation D value [6–11]. The comprehensive evaluation D value, which has the higher accuracy, has been widely used in evaluating stress tolerance in germplasms of different crops or vegetables [8, 10–15]. In this research, we investigated 10 traits related to salt tolerance and used their comprehensive evaluation D values in GWAS, which should help to improve salt tolerance evaluation of this panel and therefore the GWAS.

### Candidate QTLs/genes detected with GWAS

Salt tolerance is a important and complex trait in cotton. Molecular tagging of salt tolerance has been investigated in several previous researches with different marker types and mapping populations [7, 16–19, 43, 44]. And, a meta analysis of salt tolerance QTLs in cotton was also reported [45]. Compared to the previous reports, there was no co-location QTL with the QTLs detected herein. This low consistency may be related to these factors, including the complexity of salt tolerance mechanisms in cotton, and differences in populations, salt tolerance assessment traits, marker types, and marker densities used in different studies. In this study, a high-density SNP array and four sets of D value (three years and BLUPed D value) were used in GWAS and 13 candidate QTLs controlling salt tolerance at germination stage were detected in no less than two environments. In addition, the QTLs regions harbored candidate genes whose functions were considered to be involved in salt tolerance and regulated by salt stress.

Many candidate genes from the 13 QTLs are very likely to be associated with salt tolerance, based on their annotation in *Arabidopsis thaliana* and functional annotation (Table 3 and Additional file 8: Table S6). Eleven genes are involved in seven TF families, namely, MYB, bZIP, ERF, TALE, SBP, HD-ZIP, and ARR-B. TFs play a major role in plant biotic and abiotic stress responses [46]. MYB [46–48], bZIP [49–51], and ERF [28, 52, 53] are also known to be involved in responses of biotic and abiotic stress. The CO-like gene (*COL4*) positively regulates abiotic (salt and osmotic) stress tolerance in *Arabidopsis* through an ABA-dependent method [54]. SBP, the plant-specific TF that participates in plant development, may enhance stress tolerance of plants by the growth regulation [55]. The ARR-B gene *ARR12* (Gh_D07G0251 in this study) negatively regulates the responses of *Arabidopsis* to drought [56]. HD-ZIP TFs are involved in both development and stress (drought or salt) responses in *A. thaliana* [57, 58], rice [59], *Manihot esculenta Crantz* [60] and *Craterostigma plantagineum* [61]. Furthermore, the HD-ZIP gene (*GhHB1*) in *G. hirsutum* responds to root development, abscisic acid, and salt [62], suggesting that HD-ZIP genes play an important role in plant development and stress responses. As shown in Table 3, these 17 candidate genes detected in GWAS respond to acidic pH, heat, hydrogen peroxide, water deprivation, or hyperosmotic salinity stress or are involved in “defense response” and are highly likely to be related to salt tolerance. In addition, the gene Gh_A01G1564 responds to GA and JA, Gh_A02G1099 and Gh_A02G1100 responds to SA, Gh_D07G0251 and Gh_D07G0256 respond to cytokinin (CK), Gh_D07G0500 responds to ABA, and Gh_D10G0643 responds to auxin (IAA). Plant hormones such as GA, JA, SA, CK, ABA, and IAA play essential roles in plant adaptation to external stimuli and changes in the environment [3, 22, 63]. Those genes involved in “response to hormone stimulus” or “signaling” were concordant with the results of previous studies of expression profiling of plants under salt stress [5, 64, 65]. Gh_A07G0729 is also involved in calcium-mediated signaling, which plays an essential role in adapting to salt stress [3, 66, 67]. In addition, six genes were involved in “ion homeostasis” or “ion transport”, by which plant tolerance to salinity could be enhanced [68–70].

Furthermore, the GO terms enriched with candidate genes in this GWAS included carbohydrate metabolic process, metabolic process, hydrolase activity, regulation of transcription, transcription factor activity, and transporter activity, and were also mainly enriched in previous studies of responses to salt (NaCl) stress in cotton roots at the seedling stage [3, 21, 22, 65, 71]. Overexpression of chitinases in transgenic tobacco enhanced its tolerance to biotic (fungal and bacterial pathogens) and abiotic (salinity and heavy metals) stress [72], and accumulation of the chitinase isoforms was induced by heavy metal stress in plants [73]. In this GWAS, the enriched GO terms associated with salt stress included “chitin catabolic process” and “chitinase activity”.

### Candidate genes and pathways associated with salt tolerance in transcriptome sequencing

To date, studies on the mechanism of salt tolerance in cotton are limited, with most investigations focusing on transcriptome sequencing of salt-responsive genes and pathways. Xu et al. (2013) [65] examined variations in gene expression of roots after exposing plants to 200 mM NaCl for 3, 12, 72, or 144 h and revealed that the enriched GO terms were related to cellular components, including “intrinsic to membrane”, “cytoplasmic vesicle”, and “membrane part”. Peng et al. (2014) [3] identified DEGs in cotton leaves at 4 and 24 h post-application of salt stress (200 mM NaCl) and revealed enriched GO terms such as “transcription factor activity”, “response to stress”, and “regulation of biological process”. Guo et al. (2015) [5] reported that the transcripts upregulated in both salt-tolerant and salt-sensitive cultivars under 150 mM NaCl treatment enriched GO terms related to “response to stimulus”, “transcription factor activity”, “peroxisome”, and “proline metabolic process”. Zhang et al. (2016) [22] identified DEGs in *G. davidsonii* roots and leaves at 12, 24, 48, 96, and 144 h post-salt stress (200 mM NaCl) and identified DEGs that enriched the salt-responsive GO categories, including “response to oxidative stress”, “responses to osmotic stress”, “ion transport”, “response to various hormone stimulus”, “response to sucrose stimulus”, and “metabolic processes”. Additionally, genes related to metabolic processes were involved in “carbohydrate”, “hormone”, “protein”, “lipid”, “amino”, “oxidation reduction”, and “organic substance”. Shu et al. (2017) [21] reported that the DEGs of NaCl/CK were associated with the GO terms of “oxidation-reduction”, “oligosaccharide metabolic process”, “photosynthesis”, “thylakoid”, and “oxidoreductase activity”.

In the current study, we screened out 6640, 3878, and 6462 DEGs in the high salt-tolerance upland accession Han682 at 3, 24, and 72 h post salt stress respectively. Among all the DEGs, 562 were continuously upregulated and 307 were continuously downregulated. The GO terms enriched with continuously upregulated DEGs were related to “metabolic process”, “oxidation-reduction process”, “carbohydrate metabolic process”, “photosynthesis”, “oligosaccharide metabolic process”, “thylakoid”, and “oxidoreductase activity”, which were also identified in previous studies [5, 21, 22, 74]. The GO terms enriched with continuously downregulated DEGs included “intrinsic to membrane”, “cytoplasmic vesicle” and “membrane part”, “response to oxidative stress”, and “transporter activity”, were agree with the findings of previous studies [3, 5, 22, 65]. The GO term related to “oxidation-reduction” was enriched with both continuously up- and downregulated DEGs, suggesting that oxidation-reduction systems elicit more complex responses to stress. In addition, the GO terms related to “membrane”, “transporter activity”, and “thylakoid” were enriched with significant DEGs in both roots and leaves, suggesting that some mechanisms associated with salt tolerance may be shared in different plant tissues and organs [3, 5, 21, 22, 65].

The pathways related to salt tolerance, including flavonoid biosynthesis, starch and sucrose metabolism, plant hormone signal transduction, starch and sucrose metabolism, phenylpropanoid biosynthesis, phenylalanine metabolism, and phagosome, were also identified in previous studies on salt tolerance mechanism of roots of cotton seedlings [5, 21, 22, 74, 75]. These pathways may play an essential role in plant adaptation to stress. However, the pathways of pyruvate metabolism, galactose metabolism, and arachidonic acid metabolism are specific to the seedling stage. The pyruvate metabolism pathway was also founded in leaves with salt stress at the seedling stage [75]. The germination of cotton seeds under salt condition require a suitable physiological state such as salt ion homeostasis, sufficient energy supply, and the capacity to remove harmful substances. These specific pathways such as pyruvate metabolism may be able to improve seed survival rate at the seedling stage during salt stress, although the underlying mechanism is unclear. The pathway of flavonoid biosynthesis had the highest rich factor and was very highly significant (*P*-value < 0.0001) in the pathway enrichment of continuously upregulated DEGs. Flavonoids, which are polyphenolic secondary metabolic compounds, play an important role in growth, development, reproduction, and stress defense [76, 77]. Petrussa et al. (2013) [77] have shown that flavonoids constitute a secondary ROS-eliminating system in plants under severe or prolonged stress conditions. Petrussa et al. (2013) [77] and Fini et al. (2011) [76] suggest that the key role of vacuoles in ROS homeostasis might be mediated by flavonoids. Ma et al. (2014) [78] have investigated the expression of genes that are involved in the flavonoid pathway and the accumulation of flavonoids related to drought tolerance in wheat. Flavonoids also play key roles in defense responses against biotic stress [79].

Similar MYB-BHLH-WDR (MBW) complexes and a family of small MYB proteins (R3-MYB) have been shown to play a key role in the regulation of flavonoid biosynthesis [47, 80]. This confirmed that TFs play an essential role in the biotic and abiotic stress responses in plants. In the current study, 19 TF families that are always related to salt stress response [3, 5, 22], were continually differentially expressed (Table 4). Among these, three (ARF, ERF, and C2H2) were downregulated, and seven (C3H, CO-like, HSF, LBD, M-type_MADS, NF-YA, and NF-YB) were upregulated. The top six TFs were bZIP, MYB (MYB-related), HD-ZIP, NAC, bHLH, and HSF.

### Putative salt-responsive genes

Based on the biological process in UniProtKB and the functions, most of the 13 putative DEGs detected from association mapping and transcriptome sequencing were likely to respond to salt stress (Table 6 and Additional file 8: Table S6). Of these, eight (Gh_A01G1563, Gh_A02G1100, Gh_A07G0622, Gh_A07G0623, Gh_A12G0877, Gh_D07G0249, Gh_D07G0251, and Gh_D07G0500) were involved in “response to stress or defense response”, “signaling or response to signal factors”, or “transcription factors” and very likely related to the salt stress response (Tables 3 and 5). The eight salt-regulated DEGs were homologous to genes that are related to salinity tolerance such as Gh_A01G1563(CUT1) involved in fatty acid biosynthetic process, Gh_A02G1100 (WAK2) encoding wall-associated receptor kinase 2 [81, 82], and responding to ABA, Gh_A07G0622 (CLPB1) and Gh_A07G0623 (HSP18.2) encoding heat shock protein [3], Gh_A12G0877 (ERF2) encoding ethylene-responsive transcription factor [53, 56], Gh_D07G0249 (CHIT1) encoding chitotriosidase 1 [5, 73], Gh_D07G0251 (RR23) involved in cytokinin-activated signaling pathway [3, 22, 64], and Gh_D07G0500 (HVA22E) responding to ABA and stress [83, 84]. In addition, Gh_A03G1740 (BGAL3) is involved in carbohydrate metabolic process which is always the mainly enriched GO term in plant under salt stress [3, 5, 22]; Gh_D07G0250 is related to methylation which plays a significant role in salt tolerance in cotton [46, 85]; and Gh_D07G0263 (GAPN) is involved in glyceraldehyde-3-phosphate dehydrogenase (NADP+) (non-phosphorylating) activity which is associated with salt stress [86, 87].

**Table 6:** The biological process of the 13 putative genes related to salt stress in UniProtKB

Gene ID	Gene Name	Description	GO - Biological process in UniProtKB
Gh_A01G1563	CUT1	3-ketoacyl-CoA synthase 6	Fatty acid biosynthetic process, response to cold, response to light stimulus, unidimensional cell growth, wax biosynthetic process
Gh_A02G1100	WAK2	Wall-associated receptor kinase 2	Cell surface receptor signaling pathway, cellular water homeostasis, oligosaccharide metabolic process, response to salicylic acid, unidimensional cell growth
Gh_A03G1740	BGAL3	Beta-galactosidase 3	Carbohydrate metabolic process
Gh_A07G0622	CLPB1	Chaperone protein ClpB1	Positive regulation of translation, protein metabolic process, protein unfolding, response to heat, response to high light intensity,response to hydrogen peroxide
Gh_A07G0623	HSP18.2	18.2 kDa class I heat shock protein	Stress response
Gh_A12G0877	ERF2	Ethylene-responsive transcription factor 2	Cell division, ethylene-activated signaling pathway, induced systemic resistance, jasmonic acid mediated signaling pathway, phloem or xylem histogenesis, positive regulation of transcription, DNA-templated, response to chitin, transcription, DNA-templated
Gh_D07G0243	DDB _G0268948	Putative methyltransferase DDB_G0268948	Methylation
Gh_D07G0249	CHIT1	Chitotriosidase-1	Chitin catabolic process, immune response, neutrophil degranulation, polysaccharide catabolic process, polysaccharide digestion, response to bacterium
Gh_D07G0250	abhd17c	Alpha/beta hydrolase domain-containing protein 17C	Palmitoyl-(protein) hydrolase activity
Gh_D07G0251	RR23	Two-component response regulator ORR23	Cytokinin-activated signaling pathway, phosphorelay signal transduction system, transcription, DNA-templated
Gh_D07G0258	At4g30993	Calcineurin-like metallo-phosphoesterase superfamily protein	NA
Gh_D07G0263	GAPN	NADP-dependent glyceraldehyde-3-phosphate dehydrogenase	Glyceraldehyde-3-phosphate dehydrogenase (NADP+) (non-phosphorylating) activity
Gh_D07G0500	HVA22E	HVA22-like protein e	Flower development, hyperosmotic salinity response, negative regulation of autophagy, pollen development, response to abscisic acid, response to cold, response to water deprivation

The qRT-PCR results also indicated the expression of these putative genes, Gh_A01G1563, Gh_A07G0622, Gh_D07G0243, Gh_D07G0251, Gh_D07G0623, Gh_D07G0250, Gh_D07G0258, Gh_D07G0500, Gh_A07G0623, Gh_A03G1740, Gh_A02G1100, and Gh_D07G0249, were regulated by salt stress at the germination stage. These results provide candidate genes for further research on salt tolerance mechanism of cotton. The specific functions and molecular regulation of these genes in salt tolerance of cotton need to be further studied [88].

## Conclusions

In the current study, the salt tolerance of 196 accessions was comprehensively evaluated with the comprehensive D values of 10 salt-relevant traits. Based on this, a GWAS for salt tolerance was conducted. In GWAS, 98 candidate genes were obtained in the 13 candidate QTLs from 17 significant SNPs in at least two environments. Functional annotation revealed that 35 of the 98 candidate genes were involved in salt tolerance responses. Furthermore, transcriptome sequencing of a high salt resistant accession, Han682, at three time points after salt or control treatment were conducted to verify the results of GWAS. By combining the results of GWAS and RNA-seq, 13 putative genes were identified and the expressions of 12 of these genes were verified using qRT-PCR. These results will enhance our understanding of the molecular-genetic regulation of salt stress tolerance in cotton and aid in the the modification of salinity tolerance related traits.

## Methods

### Plant materials and SNP markers

A panel of 196 diverse upland cotton accessions, including 169 genotypes from five cotton-growing regions across China and 27 from other countries, were employed in association mapping of salt tolerance at the germination stage. All these 196 accessions were inbred for at least 3 years before use in this study. The detailed information on this panel is described in Additional file 12: Table S9 [89].

The 41,815 polymorphic SNP markers screened from 77,774 SNPs (CottonSNP80K, [90]) were applied in population structure analysis and GWAS. The 196 genotypes were divided into two subpopulations and confirmed using four methods (the UPGMA (unweighted pair group method with arithmetic means) phylogeny, PCA, STRUCTURE, and kinship matrix) (Additional file 13: Figure S4) [89]. The summary of SNPs, polymorphic information content (PIC), gene diversity, and LD decay were also calculated as described by Yuan et al. (2018) (Additional file 7: Table S5) [89].

### Salt tolerance assessment at the germination stage

Salt tolerance evaluation at the germination stage, which is the most sensitive development stage to salt stress [91], was performed in a triplex randomized block experiment in 2017, with seeds from three calendar years (2014–2016). Cotton seeds delinted by sulfuric acid were surface-sterilized with 0.1% HgCl for 15 min, then uniform-sized seeds were selected for the germination test, which was performed in an incubator at 28 ± 1 °C and 80% ± 2% relative humidity. The seeds (100 seeds) were planted in a germination box (13 × 19 × 12 cm) containing 750 g dry sand and covered evenly with 250 g dry sand above the seeds, then 250 mL 200 mmol/L NaCl solution or distilled water (as control) was added. Every replicate (treatment or control) of each genotype consisted of 200 seeds in two germination boxes.

The number of germinated seeds was recorded from the 3rd day to 10th day after sowing. Then GR, GP, GI, and VI were calculated using the following formulas: GR = $\frac{G7}{TS}\times 100\%$ and GP = $\frac{G3}{TS}\times 100\%$, where *TS* is the total number of seeds in each replicate (200 were used) and *G*_*3*_ or *G*_*7*_ is the number of total germinated seeds in two germination boxes from the first day to the third or seventh day after sowing; and GI = $\sum \frac{Gt}{Dt}$, VI = GI × *S*, where *t* is the number of days after planting, *Gt* is the number of germinated seeds at the *t*th day after sowing, *Dt* is the number of days after planting corresponding to *Gt*, and *S* is the fresh weight of a single plant seedling.

In addition to germination characteristics, several other salt tolerance-related traits were also investigated. On the 10th day, 10 plants from each germination box were randomly selected for determination of PH, SFM, SDM, RL, RFM, and RDM. The average value of two germination boxes of each replicate was used for further data analysis.

The STI for each trait was calculated using the following formula: $\mathrm{STI}=\left(Y\mathrm{non}\ \mathrm{salt}\ \mathrm{stress}\times Y\mathrm{salt}\ \mathrm{stress}\right)/{\left(\overline{Y}\mathrm{non}\ \mathrm{salt}\ \mathrm{stress}\right)}^2$, where *Y*_non-salt stress_ is the phenotypic value without salt stress, *Y*_salt stress_ is the phenotypic values under salt stress, and $\overline{Y}$_non-salt stress_ is the average phenotypic value without salt stress [92, 93].

The BLUPed STI were calculated with the nine STIs (three replications × three years) of each trait using the R lmer4 package. Then, four sets of STIs (STIs in three years and the BLUPed STIs) were used in the subsequent calculations. The weights of each principal component factor were calculated as W*i* = *Ri*/ ∑ *Ri* (*i* = 1, 2, …, n), and *R*_*i*_ means the contribution rate of the *i*th principal component factor.

Subordinate function analysis was performed as follow: U(*Xi*) = (*Xi* − *X*min)/(*X* max  − *X*min) (*i* = 1, 2, …, n), where *X*_*i*_ is the value of the *i*th principal component factor, and *X*_*min*_ and *X*_*max*_ indicate the minimum and maximum value of the *i*th principal component factor [10, 11].

The comprehensive evaluation values (D) of salt tolerance were calculated as D =  ∑ (U*i* × W*i*) (i = 1, 2, …, n) [10, 11]. Data processing was conducted with Excel (Office 365) and SPSS (version 23.0, RRID:SCR_002865).

### Association mapping

Marker-trait association was performed using the software TASSEL version 5.2.40 (RRID:SCR_012837), and the threshold value was set at *p* < 0.001 for declaring a significant marker-trait association. For each chromosome, the LD decay distance was regarded as the confidence interval for the candidate QTL detected on it. With the *G. hirsutum* AD1 genome NAU-NBI assembly v1.1 [39], candidate genes were gained for each QTL.

### RNA-seq and DEG

Based on the comprehensive evaluation of salt tolerance of the 196 genotypes, the high salt-resistant accession Han682 (namely H1) was selected for RNA-seq analysis under salt stress. After being surface-sterilized with 0.1% HgCl, seeds were sown in wet sand in germination boxes. When the radicle had grown to 2–3 mm long, seeds with uniform radicles were selected and planted in sand beds with 0.3% (weight percentage, approximately 200 mmol/L) or 0% NaCl (the control) in other germination boxes. Root tissues were harvested at 3, 24, and 72 h after planting and immediately stored in liquid nitrogen. The collected root samples were designated as S3, S24, and S72 for the salt-treated plants, and H3, H24, and H72 for the control plants, respectively. Each treatment was repeated twice. RNA-seq libraries were generated using NEBNext® Ultra™ RNA Library Prep Kit for Illumina® (New England Biolabs, Ipswich, MA, USA) following the manufacturer’s recommendations. The library preparations were sequenced on an Illumina HiSeq platform in Novogene Bioinformatics Institute, Beijing, China.

An index of the reference genome (*Gossypium hirsutum* AD1 genome NAU-NBI assembly v1.1) [39] was built using Bowtie v2.2.3 [94], and clean reads were aligned to the reference genome using TopHat v2.0.12 [95]. HTSeq v0.6.1 was employed to estimate the gene expression levels using fragments per kilobase of exon per million mapped fragments [96]. DEGs in the two conditions were identified using the DESeq R package (1.18.0) based on an adjusted *P*-value < 0.05 [97].

### GO and KEGG enrichment

The GOseq R package was employed to implement the GO enrichment analysis of DEGs [98]. GO terms with *P*-value corrected by FDR < 0.05 were regarded as significantly enriched. KOBAS software was used to test the statistical enrichment of DEGs in the KEGG pathways [99].

### qRT-PCR verification

qRT-PCR was performed to validate the expression of DEGs. Total RNAs were extracted using an OmniPlant RNA kit (DNase I). The isolated RNA (1500 ng) was first reverse-transcribed to generate cDNAs using a HiFiScript cDNA synthesis kit. The cDNAs were then used as template for qRT-PCR on an Applied Biosystems® 7500 Real-Time PCR System using an UltraSYBR Mixture. In this process, all kits and mixtures were purchased from Beijing CoWin Bioscience Co. The specific primers for the target genes were designed using Primer-BLAST in NCBI and GSP (a web-based platform for designing genome-specific primers in polyploids) [100]. The *GhUBQ7* was used as intro-reference gene [101]. All the specific primers (Additional file 14: Table S10) were synthesized by Sangon Biotech Shanghai Co., Ltd. (Shanghai, China).

## Additional files

Additional file 1: **Table S1.** Descriptive statistics of the salt tolerance index of 10 salt tolerance-related traits. (XLSX 11 kb)

Additional file 2: **Table S2.** Correlation analysis among 10 traits related to salt tolerance. (XLSX 9 kb)

Additional file 3: **Figure S1.** Screen plot of eigenvalues with component number for STIs in BLUP and 3 years. (DOCX 190 kb)

Additional file 4: **Table S3.** Characteristics, contribution rates, and weights of two principal components at the germination stage. (XLSX 9 kb)

Additional file 5: **Figure S2.** PCA plots for the STIs of 10 salt tolerance traits in BLUP and 3 years. a for 2014, b for 2015, c for 2016, and d for BLUP. (DOCX 79 kb)

Additional file 6: **Table S4.** Comprehensive evaluation values (D) and K-mean cluster analysis of salt tolerance. (XLSX 20 kb)

Additional file 7: **Table S5.** Summary of SNPs, PIC, gene diversity, and LD decay in Yuan et al. (2018) [89]. (XLSX 11 kb)

Additional file 8: **Table S6.** Annotation of genes located within candidate QTL regions. (XLSX 25 kb)

Additional file 9: **Table S7.** Summary of sequencing data quality. (XLSX 11 kb)

Additional file 10: **Figure S3.** Validation of the 20 random genes in transcriptome sequencing with qRT-PCR. (XLSX 11 kb)

Additional file 11: **Table S8.** Candidate genes near significant SNPs. (DOCX 7557 kb)

Additional file 12: **Table S9.** Information for 196 upland cotton accessions [89]. (XLSX 23 kb)

Additional file 13: **Figure S4.** Population structure of the 196 accessions in Yuan et al. (2018) [89]. (A) UPGMA tree based on Nei’s genetic distances. (B) Principal component analysis of 196 accessions based on genotype. (C) Population structure of the 196 accessions based on STRUCTURE when K = 2. (D) Kinship for this panel. (DOCX 693 kb)

Additional file 14: **Table S10.** All the specific primers for the selected genes and the intro-reference gene UBQ7. (XLSX 12 kb)

## Data Availability

Most of the data pertaining to the present study has been included in the Tables/Figures of the manuscript. The authors are pleased to share the rest of the raw data upon request.

## Abbreviations

ABA: Abscisic acid
BLUP: Best linear unbiased prediction
BP: Biological process
CC: Cellular component
CDS: Coding sequences
CV: Coefficient variation
DEGs: Differentially expressed genes
GA: Gibberellic acid
GI: Germination index
GO: Gene Ontology
GP: Germination potential
GR: Germination rate
GWAS: Genome-wide association study
JA: Jasmonic acid
KEGG: Kyoto Encyclopedia of Genes and Genomes
KMO: Kaiser-Mayer-Olkin
LD: Linkage disequilibrium
lncRNAs: Long non-coding RNAs
MF: Molecular function
MLM: Mixed linear model
PCA: Factor analysis of principal component analysis
PH: Plant height
PIC: Polymorphic information content
QQ: Quantile quantile
QTL: Qualitative-trait locus
QTLs: Qualitative-trait loci
RDM: Root dry mass
RFM: Root fresh mass
RL: Root length
RNA-seq: RNA sequencing
SA: Salicylic acid
SDM: Shoot dry mass
SFA: Subordinate function analysis
SFM: Shoot fresh mass
SNPs: Single-nucleotide polymorphisms
STI: Salt tolerance index
TF: Transcription factor
UPGMA: Unweighted pair group method with arithmetic means
VI: vVigor index
