# Supplementary figures and images for: Genome-wide association and differential expression analysis of salt tolerance in Gossypium hirsutum L at the germination stage
Source: BMC Plant Biol. 2019 Sep 11;19:394. doi: 10.1186/s12870-019-1989-2 (PMC6737726; doi:10.1186/s12870-019-1989-2)

**
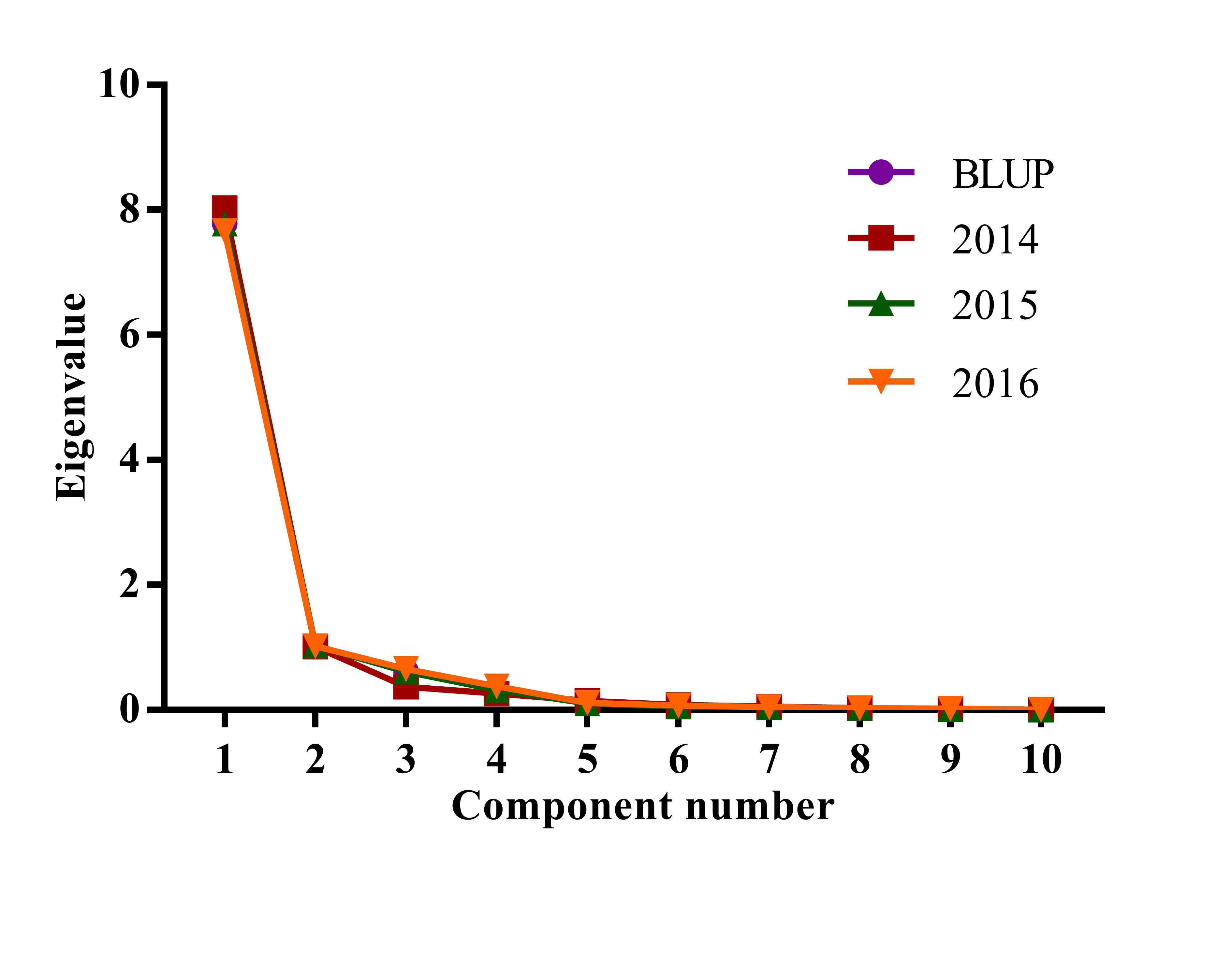
Figure S1** **Screen plot of eigenvalues with component number for STIs in BLUP and three years.**

Supplement: Supplementary file 3 — Figure S1. Screen plot of eigenvalues with component number for STIs in BLUP and 3 years. (DOCX 190 kb) [file 12870_2019_1989_MOESM3_ESM.docx]

**
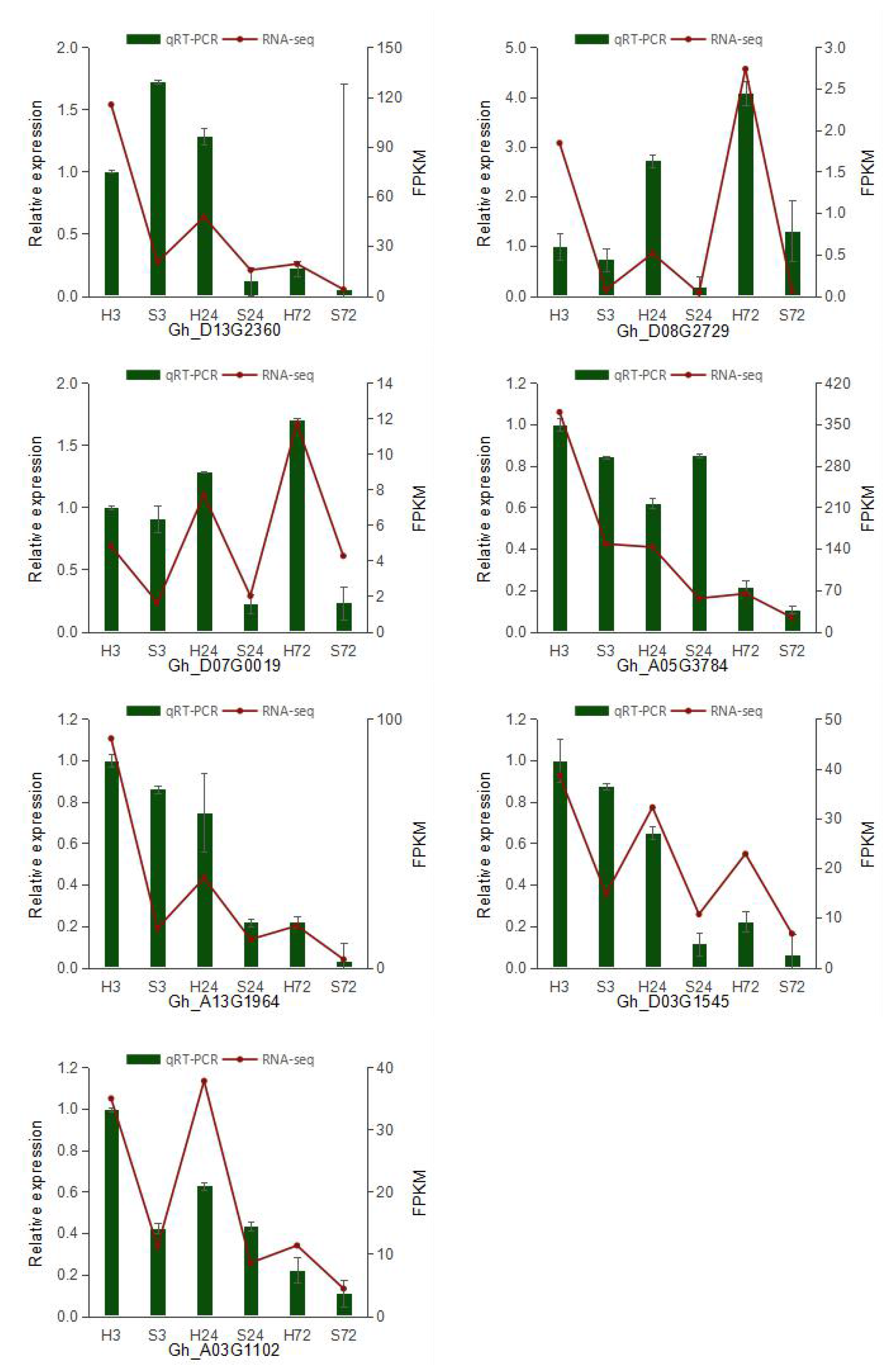
**

**
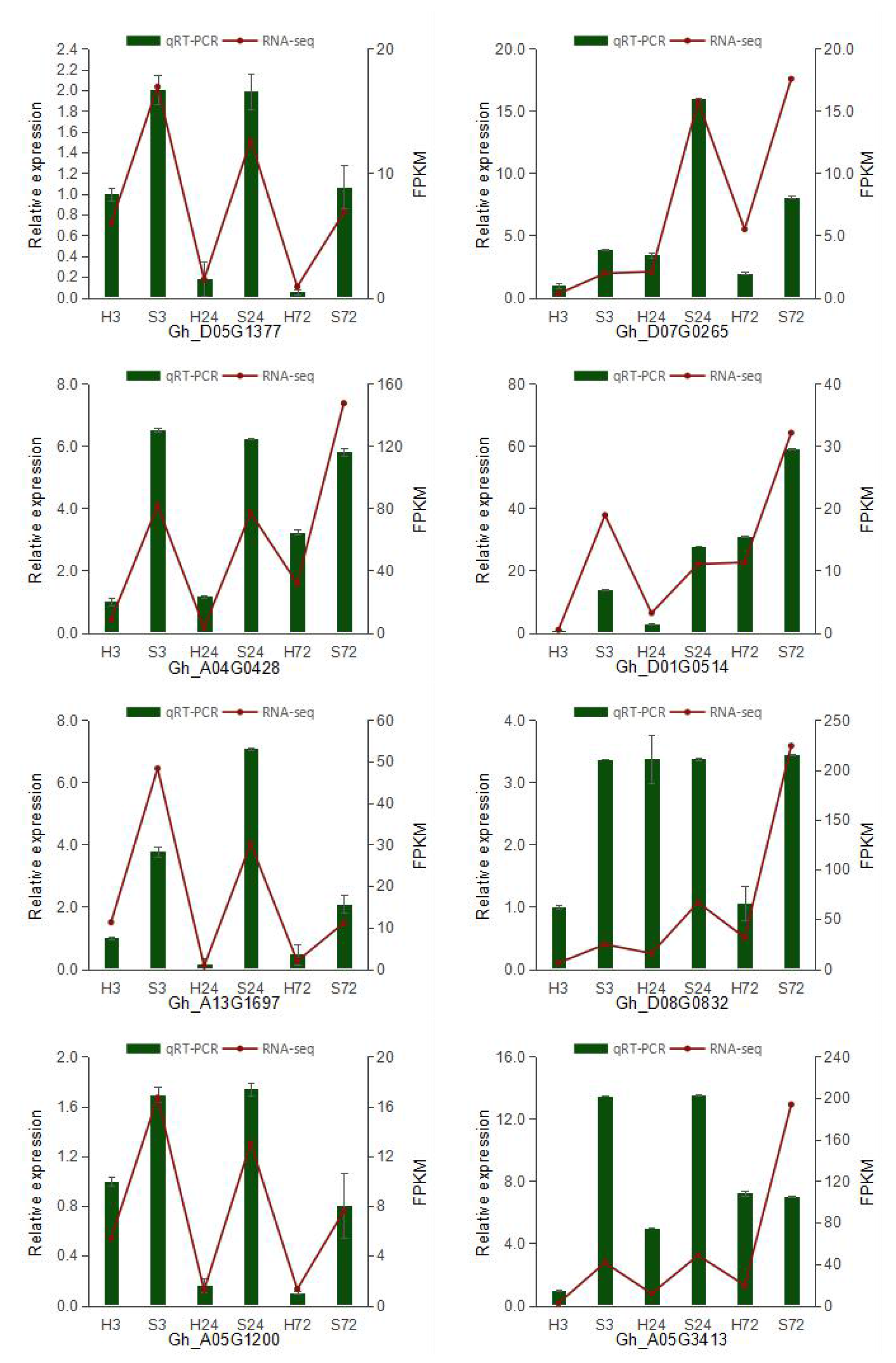
**

**
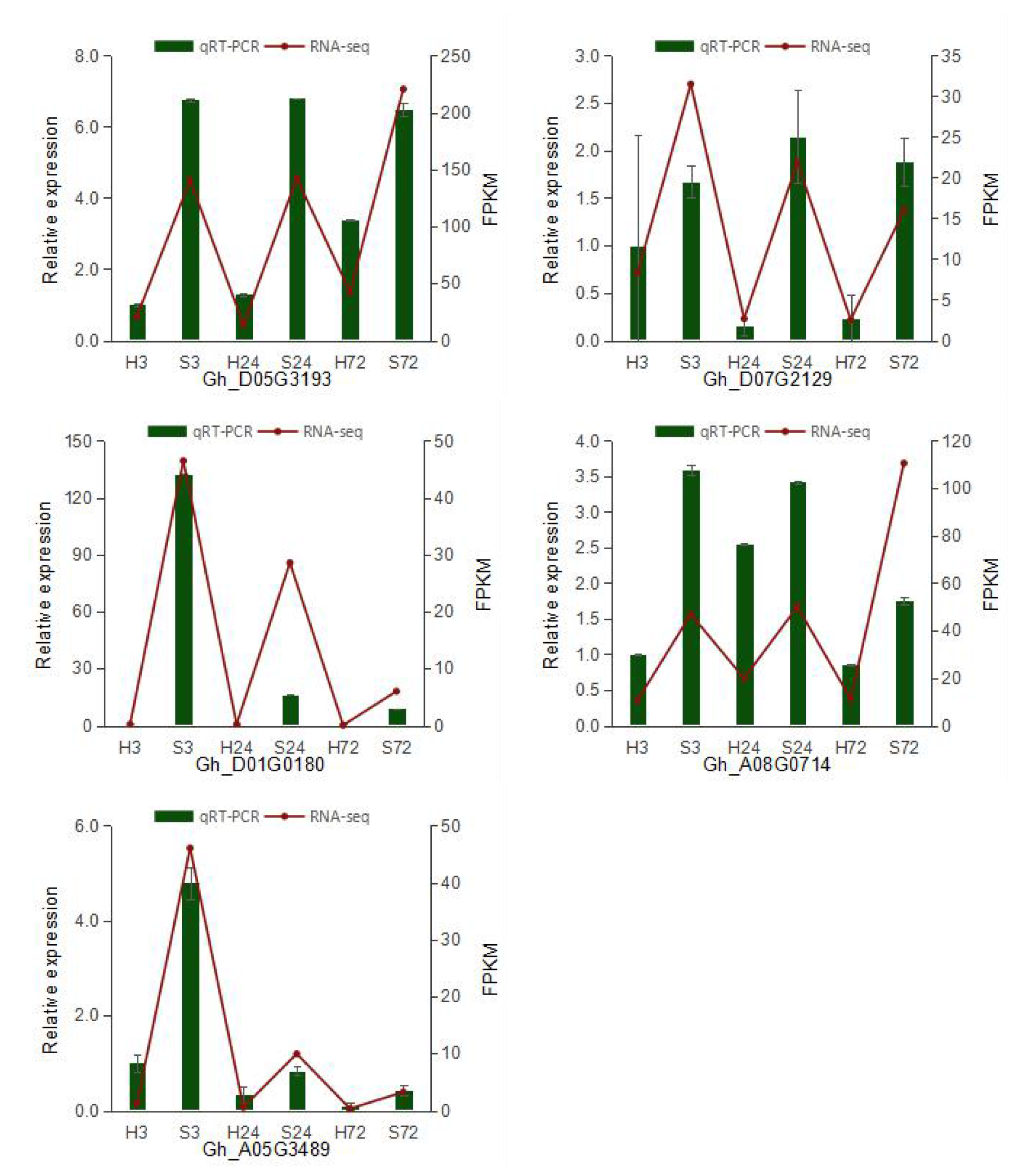
Figure S3 Validation of the 20 random genes in transcriptome sequencing with qRT-PCR.**

Supplement: Supplementary file 11 — Table S8. Candidate genes near significant SNPs. (DOCX 7557 kb) [file 12870_2019_1989_MOESM11_ESM.docx]
