# Supplementary material for: Genome-wide association and differential expression analysis of salt tolerance in Gossypium hirsutum L at the germination stage
Source: BMC Plant Biol. 2019 Sep 11;19:394. doi: 10.1186/s12870-019-1989-2 (PMC6737726; doi:10.1186/s12870-019-1989-2)
Supplement: Supplementary file 5 — Figure S2. PCA plots for the STIs of 10 salt tolerance traits in BLUP and 3 years. a for 2014, b for 2015, c for 2016, and d for BLUP. (DOCX 79 kb) [file 12870_2019_1989_MOESM5_ESM.docx]

**
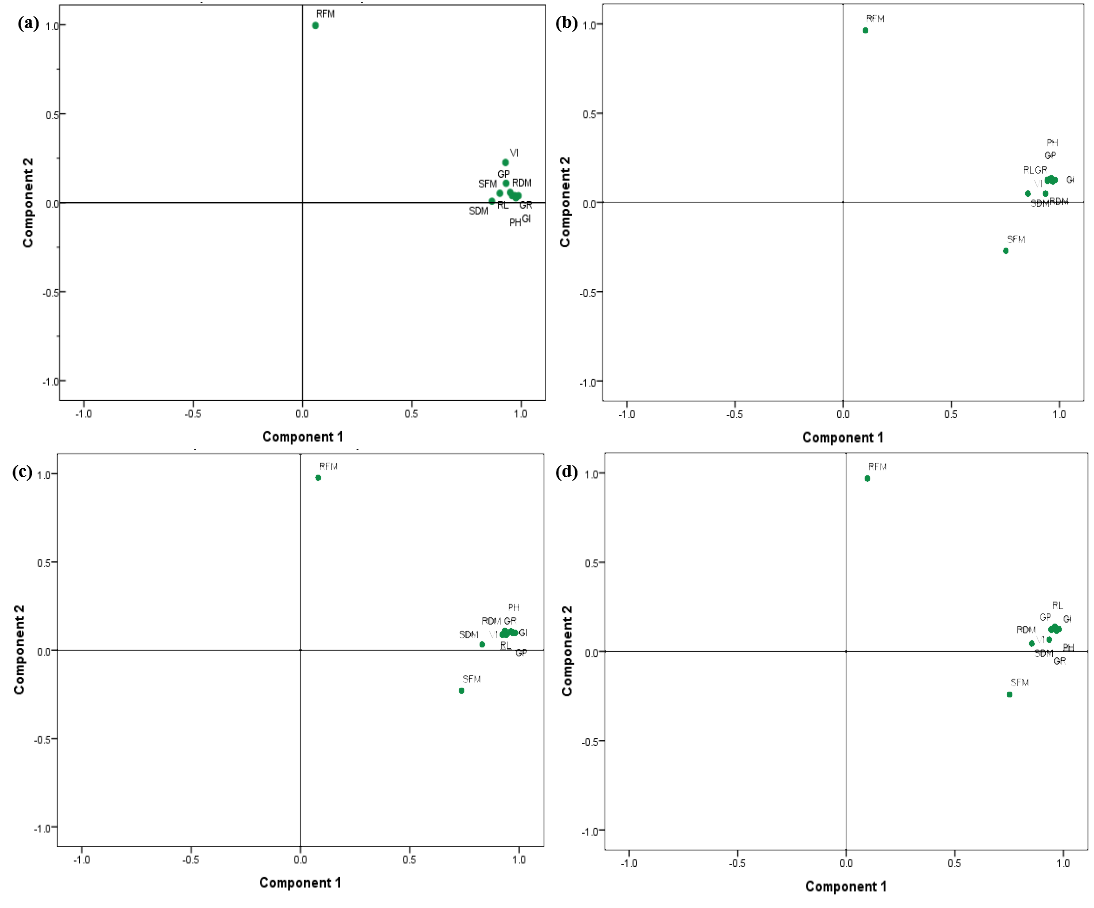
Figure S2 PCA plots for the STIs of ten salt-tolerance traits in BLUP and three years. a for 2014, b for 2015, c for 2016 and d for BLUP.**
