# Supplementary material for: Genome-wide association and differential expression analysis of salt tolerance in Gossypium hirsutum L at the germination stage
Source: BMC Plant Biol. 2019 Sep 11;19:394. doi: 10.1186/s12870-019-1989-2 (PMC6737726; doi:10.1186/s12870-019-1989-2)
Supplement: Supplementary file 13 — Figure S4. Population structure of the 196 accessions in Yuan et al. (2018) [89]. (A) UPGMA tree based on Nei’s genetic distances. (B) Principal component analysis of 196 accessions based on genotype. (C) Population structure of the 196 accessions based on STRUCTURE when K = 2. (D) Kinship for this panel. (DOCX 693 kb) [file 12870_2019_1989_MOESM13_ESM.docx]

**
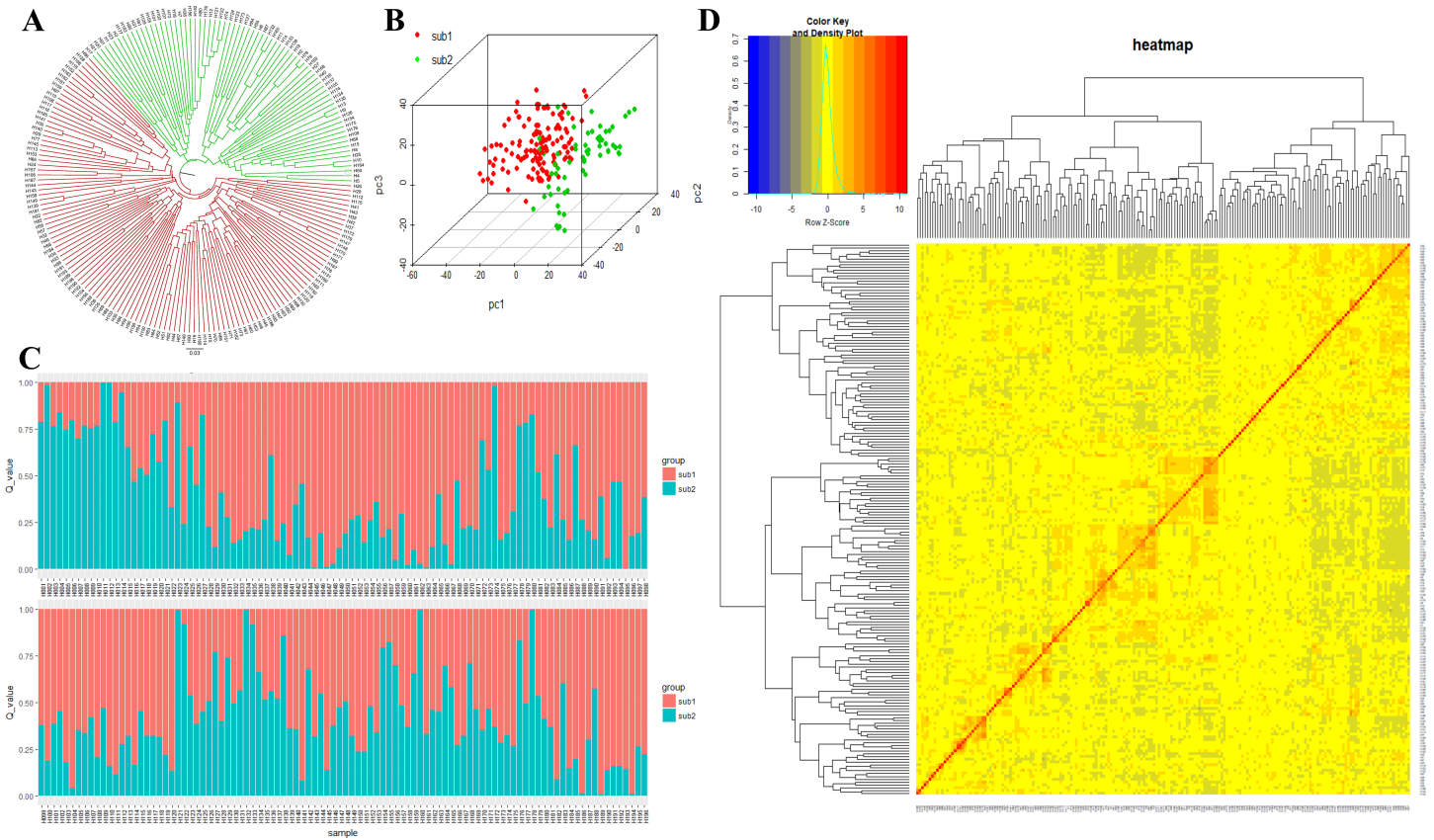
Figure S4 Population structure of the 196 accessions in Yuan et al. (2018) [89].** (**A**) UPGMA tree based on Nei’s genetic distances. (**B**) Principal component analysis of 196 accessions based on genotype. (**C**) Population structure of the 196 accessions based on STRUCTURE when K = 2. (**D**) Kinship for this panel.
